# Robot-assisted kidney transplantation in living and deceased donors: a six-year experience

**DOI:** 10.1007/s11701-026-03773-z

**Published:** 2026-08-11

**Authors:** Hafiz Umair Siddiqui, Dylan Isaacson, Sami Shoucair, Wei Wu, Venkatesh Krishnamurthi, Yi-Chia Lin, Alvin Wee, Jihad Kaouk, Mohamed Eltemamy

**Affiliations:** 1 Department of Urology, Glickman Urological and Kidney Institute, Cleveland Clinic, 9500 Euclid Avenue, NA-23, OH 44195-3733 Cleveland, USA; 2 Urology Department of Kasr Alaini, Cairo University, Cairo, Egypt

**Keywords:** Robot-Assisted Kidney transplantation, RAKT, Living donor, Deceased donor, Open surgery, Perioperative outcomes, Robotic Surgery, Single-Port Robotic Transplant, Multi-port Robotic Transplant

## Abstract

Robot-assisted kidney transplantation (RAKT) is increasingly adopted, but reports describing large-volume programs with predominantly deceased-donor transplantation, increasing procedural complexity, and experience across both single-port (SP) and multiport (MP) robotic platforms remain limited. In this retrospective, single-center study, we reviewed a prospectively maintained registry of patients who underwent RAKT between October 2019 and December 2025 and evaluated perioperative, postoperative, and allograft outcomes. A total of 228 patients underwent RAKT, including 125 (54.8%) deceased-donor kidney transplants (DDKT) and 103 (45.2%) living-donor kidney transplants (LDKT). SP and MP platforms were utilized in 30.7% and 69.3% of cases, respectively. Six patients received dual-kidney transplants and four underwent simultaneous kidney-pancreas transplantation. Annual case volume increased from one procedure in 2019 to 115 procedures in 2025. Median operative times were 3 h 38 min for DDKT and 4 h 6 min for LDKT, with median vascular anastomosis times of 44 and 40 min, respectively. Conversion to open surgery occurred in one patient (0.4%). Median length of stay was four days for DDKT and two days for LDKT. Delayed graft function occurred in 30.4% of DDKT recipients and 4.8% of LDKT recipients; among the 121 recipients with available one-year serum creatinine, median one-year serum creatinine was 1.49 mg/dL and 1.37 mg/dL, respectively. Graft-loss occurred in two DDKT recipients (1.6%) and one LDKT recipient (0.9%), while three deaths (2.4%) occurred after DDKT and one death (0.9%) after LDKT. In this large single-center experience, RAKT was associated with low conversion rates, favorable perioperative outcomes, and good one-year graft function despite increasing procedural complexity, supporting its continued expansion in appropriately selected patients.

## Introduction

Kidney transplantation is the optimal treatment for patients with end-stage renal disease (ESRD) [1, 2]. Robot-assisted kidney transplantation (RAKT) has been increasingly adopted over the last two decades [3–5]. Robotic platforms provide enhanced visualization and greater precision, potentially reducing the technical challenges associated with open transplantation, especially in the obese patient population [6–8]. Prior studies have demonstrated the safety and feasibility of RAKT in select patient populations, with perioperative outcomes and graft function comparable to open kidney transplantation [8–13]. However, the existing literature largely describes outcomes from living donor kidney transplants (LDKT), whereas research into robotic DDKT has been relatively neglected [14–19].

Here we report our experience with RAKT over six years as we introduced both single-port and multi-port robotic systems (Intuitive Surgical Inc., Sunnyvale, CA, USA). The primary aim of this work was to describe our institutional experience with RAKT, including its safety, feasibility, and evolution over time. Secondary aims included describing outcomes by donor type, tracing temporal trends in use of single-port and multi-port platforms and characterizing our institutional learning curves. We show the growth of our program from our initial case to one of the highest annual RAKT volumes worldwide. We demonstrate good clinical outcomes while performing increasingly complex cases—including dual kidneys, simultaneous kidney/pancreas transplants, kidneys with multiple arteries/arterial reconstruction, re-transplants and recipients with calcified arteries. Finally, we believe this to be the largest experience with single port extraperitoneal RAKT, which, to our knowledge, no other centers have described [20]. 

## Materials and methods

### Study design and patient population

*Ethical Considerations*: This study was approved by the Institutional Review Board at the Cleveland Clinic Foundation.

This is a retrospective analysis of our prospectively maintained Robotic Kidney Transplant (RoKiT) registry. Patients ≥ 16 years who underwent living or deceased donor RAKT between October 2019-December 2025 were included. We performed our first RAKT in October 2019 using the SP robotic platform. We subsequently introduced the MP robot in 2023 after 32 SP cases.

Among patients undergoing RAKT, the choice between SP and MP techniques depended on robot availability and anatomical complexity. SP RAKT was preferred in cases with relatively smaller grafts, living donor kidneys, single renal vessels, and minimal iliac vessel calcifications, whereas MP RAKT was favored for larger grafts, deceased donor kidneys, multiple renal vessels and significant recipient arterial calcifications.

The study cohort was stratified by donor type (deceased vs. living). Most procedures were performed by a single primary surgeon, with a second primary surgeon contributing beginning in 2025.

## Surgical technique

### Back table kidney preparation

A standardized back-table bench preparation protocol was developed for use in SP and MP robotic kidney transplants. Perinephric fat is removed using a bipolar vessel sealing device (LigaSure, Medtronic, Minneapolis, MN, USA). The renal vessels are cleared off and marked to maintain orientation during implantation. The ureter is trimmed and spatulated, and a ureteral stent is pre-placed. The allograft is enclosed in a blue surgical glove to allow for atraumatic introduction into the operative field and easier orientation/graft handling.

## Single-port robotic technique

Our single-port RAKT technique has been described previously [21]. The patient is positioned supine, and a single 5-cm Pfannenstiel or lower midline incision is performed, based on the patient’s anatomy and prior surgical history. The extraperitoneal space is developed bluntly allowing for placement of the SP bubble port. A floating dock technique is then utilized, allowing adequate instrument triangulation and working distance within the extraperitoneal space [22]. We used ice slush to attain regional hypothermia in our first ten cases, after which we did not employ regional hypothermia techniques due to improved anastomotic times.

## Multi-port robotic technique

Multiport RAKT is performed using a transperitoneal approach with the patient in the Trendelenburg position. A Pfannenstiel or lower midline incision is utilized to gain access to the peritoneal cavity. Four additional 8-mm robotic ports are placed above the level of the umbilicus, triangulating the side of implantation, and the robotic system is docked from the patient’s side.

**Fig. 1:**
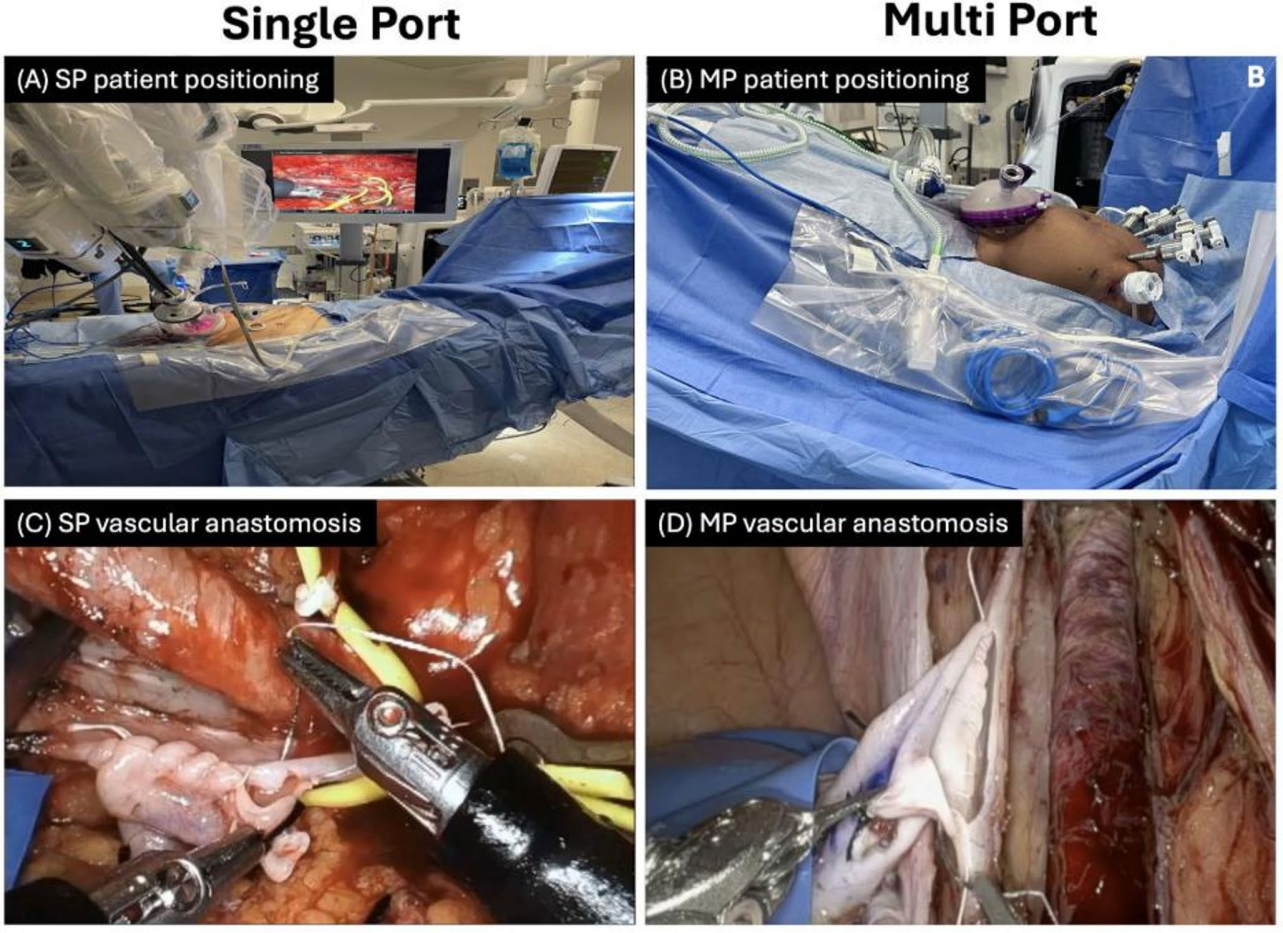
Patient Positioning and Vascular Anastomosis in Single-Port and Multi-Port Robot-Assisted Kidney Transplantation. (**A**) Patient positioning for single-port (SP) robot-assisted kidney transplantation. (**B**) Patient positioning for multi-port (MP) robot-assisted kidney transplantation. (**C**) Venous anastomosis between the renal vein and the external iliac vein using the SP robot. (**D**) Venous anastomosis between the renal vein and the external iliac vein using the MP robot.

For both SP and MP RAKT, the external iliac vessels are circumferentially dissected. The vascular anastomoses are performed in an end to side fashion using running 6 − 0 Gore-Tex^®^ suture (W.L. Gore & Associates, Flagstaff, AZ, USA), with smaller supernumerary arteries anastomosed using 7 − 0 Prolene suture (Ethicon, Somerville, NJ, USA). A refluxing ureteroneocystostomy is performed using running 4 − 0 Vicryl suture (Ethicon, Somerville, NJ, USA) for both SP and MP transplants. Key steps of SP and MP RAKT are illustrated in Fig. 1.

### Data collection

Recipient demographics, intraoperative/postoperative data, and clinical outcomes were extracted. Postoperative pain scores were assessed by inpatient nursing staff using a 1–10 scale and were retrospectively extracted from the electronic medical record for analysis. The degree of external iliac artery (EIA) calcifications on preoperative CT was graded based on morphology and thickness as described by Davis et al. [23].

Cold ischemia time in both living and deceased donors was defined as the interval from initiation of cold preservation until removal of the kidney from ice immediately before implantation. Donor warm ischemia time refers to arterial clamping to initiation of cold perfusion in living donors and functional donor warm ischemia time in DCD donors. Anastomosis time was defined from the start of the first vascular anastomosis until graft reperfusion.

Delayed graft function (DGF) was defined as dialysis within one week after transplantation. Renal function was assessed using serum creatinine at hospital discharge, 3 months, 1 year, and the last available point of follow-up.

### Statistical analysis

Baseline demographic and clinical characteristics were compared between living and deceased donor recipients using Student’s t-test, Mann–Whitney U test, Chi-Square test, or Fisher’s exact test. Given the differences between our living and deceased donor cohorts, perioperative and postoperative outcomes were reported as medians and interquartile ranges, without direct statistical comparisons. Data completeness was reviewed before analysis. Cases with missing values were excluded only from analyses requiring those specific variables.

Learning curve analysis was performed using cumulative sum (CUSUM) methodology. The overall median operative time was used as the reference value, and the analysis included consecutive recipients with a single renal artery. A standard CUSUM approach without predefined acceptable or unacceptable performance thresholds was used. Donor type, robotic platform, case complexity, and trainee involvement were not incorporated into the CUSUM model. A second primary surgeon joined the program in 2025 and affected only this portion of the learning curve. Locally estimated scatterplot smoothing (LOESS) curves were generated to illustrate temporal trends in operative and vascular anastomosis times [24]. Analyses were conducted using JASP version 0.19.3 (Amsterdam, Netherlands) or R statistical software version 4.5.2. (R Foundation, Vienna, Austria).

## Results

### Case volumes

**Fig. 2:**
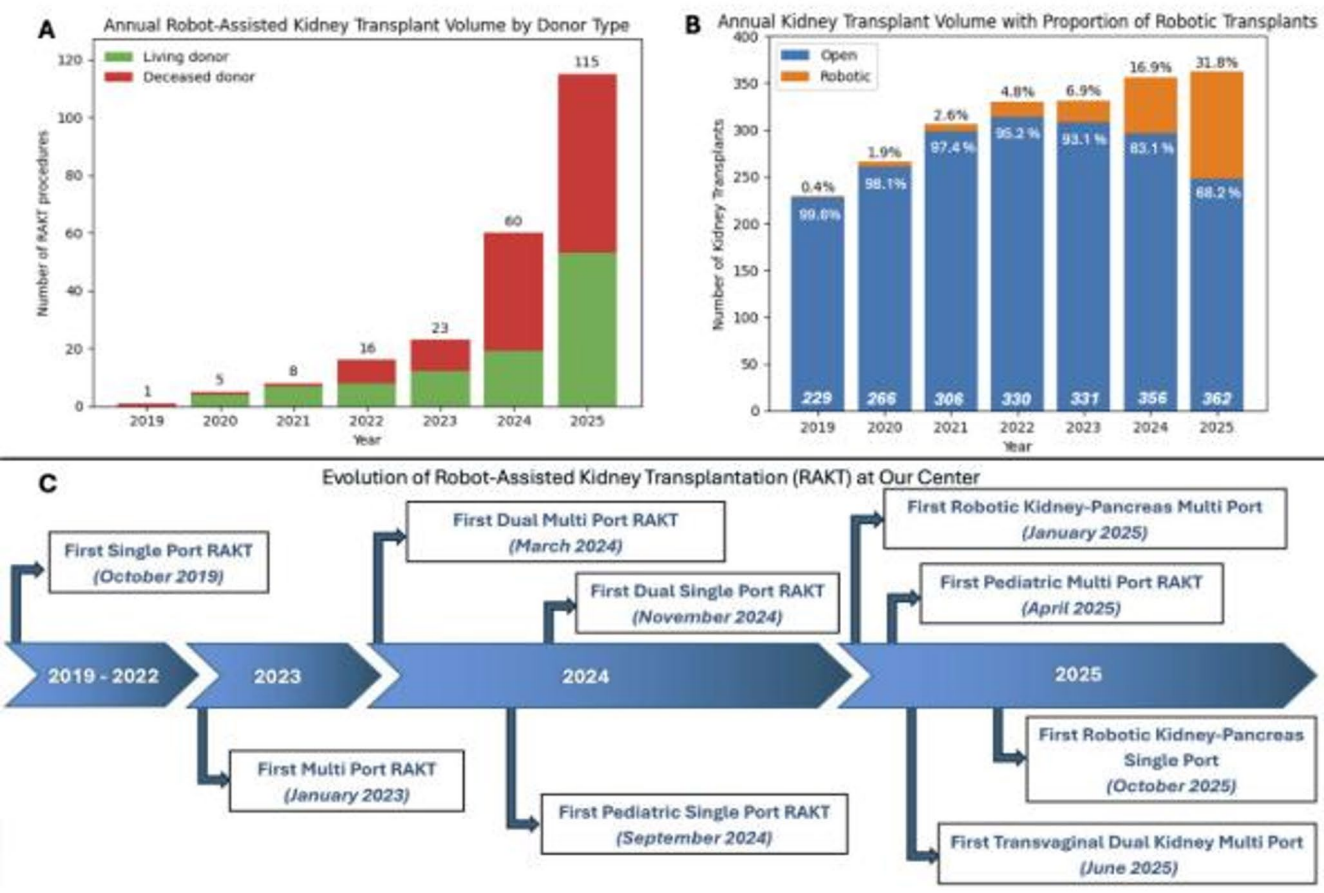
Growth of robotic volume over time illustrated using stacked bar graphs: (**A**) Annual robot-assisted kidney transplantation (RAKT) volume broken down by donor type (deceased vs. living). (**B**) Annual center kidney transplant volume demonstrating the proportion of RAKT vs. Open kidney transplants. (**C**) Schematic timeline illustrating the evolution of robot-assisted kidney transplantation (RAKT) at our institution.

228 patients underwent RAKT at our institution during the study period. Of these, 125 (54.8%) received DDKT and 103 (45.2%) received LDKT. Annual RAKT volume increased steadily over the study period, with an increasing proportion of our total volume performed robotically compared to open (Fig. 2).

### Recipient demographics and preoperative characteristics

Baseline demographics are presented in Table 1. DDKT recipients were older (median 58.9 vs. 50.4 years, *p* < 0.001), more frequently African American (49.6% vs. 5.8%, *p* < 0.001) more commonly received Right sided kidneys (40.8% vs. 11.7%, *p* < 0.001), and had higher median BMI (33.4 vs. 30.3 kg/m², *p* = 0.003). Median Kidney Donor Profile Index (KDPI) for DDKT recipients was 55% (IQR: 38–74%).

**Table 1:** Baseline Demographics and Preoperative Clinical Characteristics

Characteristics	Overall cohort *n* = 228	Deceased donor (*n* = 125)	Living donor (*n* = 103)	*p* value
Recipient age, years *median (IQR)*	56.6 (45.2–64.3)	58.9 (50-67.3)	50.4 (41.6–61.2)	< 0.001
Body mass index, kg/m² *median (IQR)*	31.7 (27.95–36.83)	33.4 (28.4–37.6)	30.3 (25.6–34.5)	0.003
Race, *n (%)* • African American • White • Other	68 (30%) 143 (62.7%) 17 (7.3%)	62 (49.6%) 54 (43.2%) 9 (7.2%)	6 (5.8%) 89 (86.4%) 8 (7.78%)	< 0.001
Sex, Male *n (%)*	149 (65.4%)	83 (66.4%)	66 (64%)	0.71
Preemptive, *n (%)*	88 (38.6%)	34 (27.2%)	54 (52.4%)	< 0.001
Months on dialysis, *median (IQR)*	21.9 (10.5–46.9)	34.7 (14.9–56.7)	12.05 (6.2–12.9)	< 0.001
Previous kidney transplant, *n %)*	13 (4.5%)	6 (4.8%)	7 (6.7%)	0.79
Laterality of kidney used *n (%)* • Left • Right • Dual • Left with Pancreas • Right with Pancreas	155 (67.9%) 63 (27.6%) 6 (2.6%) 2 (0.9%) 2 (0.9%)	64 (51.2%) 51 (40.8%) 6 (4.8%) 2 (1.6%) 2 (1.6%)	91 (88.3%) 12 (11.7%) n/a n/a n/a	< 0.001
Karnofsky Performance Score (%) *median (IQR)*	80 (70–90)	80 (70–90)	80 (70–90)	0.96
Diabetes, *n (%)*	90 (39.4%)	65 (52%)	25 (24.2%)	< 0.001
Hypertension, *n (%)*	189 (85.5%)	105 (84%)	84 (81.5%)	0.21
Peripheral vascular disease, *n (%)*	19 (8.3%)	13 (10.4%)	6 (5.8%)	0.12
EIA calcification, *n (%)* • Grade 1 • Grade 2 • Grade 3	53 (23.2%) 18 (7.8%) 13 (5.7%) 22 (9.6%)	41(32.8%) 14 (11.2%) 10 (8%) 17 (13.6%)	12 (11.6%) 4 (3.9%) 3 (2.9%) 5 (4.9%)	< 0.001
Number of arteries in donor graft, n *(%)* • 1 • 2 • 3	191 (83.8%) 33 (14.5%) 4 (1.7%)	101 (81%) 22 (17.6%) 2 (1.6%)	90 (87.4%) 11 (10.7%) 2 (1.9%)	0.33
Number of arterial anastomoses, *n (%)* • 1 • 2	204 (89.4%) 24 (10.5%)	113 (90.4%) 12 (9.6%)	91(88.3%) 12 (11.7%)	0.11
Use of 3rd party cadaveric donor graft *n (%)*	8 (3.5%)	6 (4.8%)	2 (1.9%)	0.22
Previous abdominal surgery, *n (%)*	107 (46.9%)	62 (49.6%)	45 (43.6%)	0.37
Deceased type, *n (%)* • DBD • DCD	42 (33.6%) 83 (66.4%)	42 (33.6%) 83 (66.4%)	n/a	
KDPI (%), *median (IQR)*	55 (38–74)	55 (38–74)	n/a	
Cause of ESRD, *n (%)* Diabetes Hypertension Glomerular disease ADPKD Retransplant Other	68 (29.8%) 42 (18.5%) 50 (21.9%) 23 (10.1%) 13 (5.7%) 32 (14%)	50 (40%) 28 (22.4%) 19 (15.2%) 9 (7.2%) 6 (4.8%) 13 (10.4%)	18 (17.5%) 14 (13.6%) 31 (30.1%) 14 (13.6%) 7 (6.8%) 19 (18.4%)	0.40

EIA, external iliac artery; KDPI, Kidney Donor Profile Index; DBD, donation after brain death; DCD, donation after circulatory death

LDKT recipients were more frequently transplanted preemptively (54% vs. 34%, *p* < 0.001). Deceased donor recipients had longer dialysis vintage prior to transplantation (median 34.7 months vs. 12 months, *p* < 0.001).

sex distribution, prevalence of diabetes and hypertension, pretransplant Karnofsky Performance Status (KPS), and history of prior abdominal surgery were similar between groups. Vascular conduits from third-party cadaveric donors were used for reconstruction in six (4.8%) DDKT and two (1.9%) LDKT. External iliac calcifications on CT were more prevalent among DDKT recipients (32.8% vs. 11.6%, *p* < 0.001).

### Operative characteristics

Intraoperative characteristics are summarized in Table 2. The SP robot was used in 16% of DDKT and 48.5% of LDKT. Four recipients had kidneys introduced transvaginally, including three DDKT recipients and one LDKT recipient. Among the DDKT group, 6 dual kidney transplants and 4 simultaneous kidney-pancreas transplants were performed. Median overall operative time was 3 h, 38 min for DDKT and 4 h, 6 min for LDKT. Median vascular anastomosis time was 44 min (IQR: 36–53) for DDKT and 40 min (IQR: 31–50) for LDKT. Median cold ischemia time was 21 h, 4 min (IQR: 15:04–26:53) for DDKT and 1 h, 30 min (IQR: 01:12 − 01:48) for LDKT. Median estimated blood loss was 50 mL in both cohorts.

**Table 2:** Intraoperative Metrics and Postoperative Clinical Outcomes

Variables	Overall cohort (*n* = 228)	Deceased donor (*n* = 125)	Living donor (*n* = 103)
*Intraoperative Characteristics*			
Robotic platform used, *n (%)* • Multiport • Single Port	158 (69.3%) 70 (30.7%)	105 (84.0%) 20 (16.0%)	53 (51.5%) 50 (48.5%)
Total Operative time, *hh: min median (IQR)*	03:52 (03:17 − 04:24)	03:38 (03:05 − 04:20)	04:06 (03:29–04:40)
Anastomosis time (single renal artery) *min median (IQR)*	42 (33–51)	44 (36–53)	40 (31–50)
Cold ischemia time, *hh: min median (IQR)*	12:37 (01:11–22:05)	21:04 (15:04–26:53)	1:30 (01:12 − 01:48)
Warm ischemia time, *min median (IQR)*	4 (3–19)	19 (0–27)	4 (3–5)
Pump used, *n (%)*	106 (46.4%)	106 (84.8%)	n/a
Pump time, *hh: min median (IQR)*	12:53 (04:24 − 18:47)	12:53 (04:24 − 18:47)	n/a
Transvaginal Kidney Introduction, *n (%)*	4 (1.7%)	3 (2.4%)	1 (0.97%)
Estimated blood loss, mL *median (IQR)*	50 (50–100)	50 (50–100)	50 (50–75)
Conversion to Open, *n (%)*	1 (0.4%)	0 (0%)	1 (0.97%)
*Post operative Outcomes*			
Length of hospital stay, *(Days) median (IQR)*	3 (2–4)	4 (3–5)	2 (2–3)
Pain (Visual Analogue Scale), *median (IQR)* • Day 1 • Day 2 • Day 3 • Day 4	6 (4–8) 5 (4–8) 4 (2–7) 4 (2–6)	7 (5–8) 6 (4–8) 4 (2–7) 4 (2–7)	6 (4–8) 5 (3–7) 3 (2–6) 4 (3–5)
DGF, *n (%)*	43 (18.8%)	38 (30.4%)	5 (4.8%)
Change in hemoglobin from admission to discharge (ΔHb), g/dL *median (IQR)*	0.9 (0.1–1.8)	1.05 (0.08–1.90)	0.8 (0.10–1.60)
Blood transfusion during the index hospitalization, *n (%)*	41 (18%)	29 (23%)	12 (11.6%)
Serum creatinine, *mg/dL median (IQR)* • Discharge • 3 months • 1 year • Last follow up	3.56 (2.06–6.60) 1.50 (1.27–1.90) 1.45 (1.22–1.70) 1.45 (1.20–1.83)	6.01 (4.15–8.43) 1.71 (1.33–2.15) 1.49 (1.31–1.81) 1.55 (1.30–2.15)	2.09 (1.53–2.85) 1.44 (1.20–1.64) 1.37 (1.16–1.62) 1.34 (1.13–1.56)
Median follow-up months *median (IQR)*	9.03 (4.2–20.13)	9.9 (4.4–17.1)	8.0 (3.6–26.9)
*Complications*			
3*0-Day complications*			
ICU admission, *n (%)* • Hypertension • Hypotension • Respiratory distress • Difficulty to extubate • Other	35 (15.4%) 10 (4.3%) 7 (3%) 9 (3.9%) 5 (2.2%) 4 (1.8%)	31 (24.8%) 9 (7.2%) 6 (4.8) 8 (6.4%) 5 (4%) 3 (2.4%)	4 (3.9%) 1(1%) 1 (1%) 1 (1%) 0 (0%) 1 (1%)
DVT, *n (%)*	2 (0.9%)	2 (1.6%)	0 (0%)
Bleeding requiring reoperation, *n (%)*	3 (1.3%)	1 (0.8%)	2 (1.9%)
Wound complication, *n (%)*	5 (2.2%)	3 (2.4%)	2 (1.9%)
30-day Readmission, *n (%)*	66 (28.9%)	40 (32%)	26 (25.2%)
*Post transplant Complications – After 30 Days*:			
Lymphocele (drainage), *n (%)*	2 (0.9%)	0 (0%)	2 (1.9%)
Acute rejection on biopsy, *n (%)*	9 (3.9%)	6 (4.8%)	3 (2.9%)
Ureteric stricture, *n (%)*	3 (1.3%)	2 (1.6%)	1 (0.9%)
Death-censored graft loss during follow up *n (%)*	3 (1.3%)	2(1.6%)	1(0.9%)
1-year Mortality, *n (%)*	4 (1.7%)	3 (2.4%)	1 (0.9%)

SP = single-port MP = multi-port DGF = delayed graft function ICU = intensive care unit DVT = deep vein thrombosis POD = postoperative day IQR = interquartile range (ΔHb = change in hemoglobin

One transplant (0.4%) required conversion from SP robotic to open. Following completion of the vascular anastomoses, the kidney appeared soft despite the presence of arterial pulse. As a precaution, the case was converted by extending the midline incision, and the arterial anastomosis was redone with subsequent improvement in graft perfusion and consistency.

### Postoperative Outcomes

Postoperative outcomes are summarized in Table 2. Median length of hospital stay was four days (IQR: 3–5) for DDKT recipients and two days (IQR: 2–3) for LDKT recipients. Median postoperative pain scores, measured using a 1–10 visual analog scale, were 7/10 on postoperative day (POD) 1 and 6/10 on POD2 in the DDKT group, and 6/10 on POD1 and 5/10 on POD2 in the LDKT group.

23% of DDKT recipients and 11.6% of LDKT recipients received a blood transfusion during their index hospitalization. The change in hemoglobin (ΔHb) from admission to discharge was 1.05 g/dL for DDKT and 0.80 g/dL for LDKT. DGF occurred in 30.4% of DDKT recipients and 4.8% of LDKT recipients.

### Renal function outcomes

Renal function outcomes are summarized in Table 2.

Median follow-up was 9.9 months for DDKT and 8 months for LDKT. Median three-month serum creatinine was 1.71 mg/dL and 1.44 mg/dL for DDKT and LDKT recipients, respectively. 121 patients in the cohort reached one year of follow-up. In these patients, one-year serum creatinine was 1.49 mg/dL for DDKT recipients vs. 1.37 mg/dL in LDKT recipients.

### Postoperative complications

Postoperative complications are summarized in Table 2. Rates of ICU admission were 24.8% in DDKT and 3.8% in LDKT. In DDKT recipients, this was most frequently due to hemodynamic instability—hypo- or hypertension (15/125, 12.0%) and respiratory distress (8/125, 6.4%). Wound complications were noted in 3 DDKT recipients (3/125, 2.4%) and 2 LDKT recipients (2/103, 1.9%), while acute bleeding requiring reoperation occurred in 1/125 (0.8%) DDKT and 2/103 (1.9%) LDKT. Deep vein thrombosis occurred in two DDKT patients (1.6%). Lymphocele requiring drainage occurred only in the LDKT group (2/103, 1.9%), both following extraperitoneal single-port RAKT. Ureteric strictures were observed in two DDKT patients (1.6%) and one LDKT patient (1.0%). Hospital readmissions occurred in 40 DDKT recipients (32.0%) and 26 LDKT recipients (25.2%). There were two (1.6%) death-censored graft losses in DDKT recipients and one (0.9%) in a LDKT recipient.

We had three patient deaths within one-year post-transplant in the DDKT group (3/125, 2.4%) and one death occurred in a LDKT recipient (1/103, 0.9%). One DDKT recipient died at home 11 months post-transplant, presumably from gastrointestinal hemorrhage. A second patient died at 6 months, after a prolonged ICU readmission due to infections and respiratory failure. The third DDKT patient died two weeks post-transplant due to acute intra-abdominal hemorrhage. One LDKT recipient died on POD11 due to a bowel perforation.

### Learning curve

**Fig. 3:**
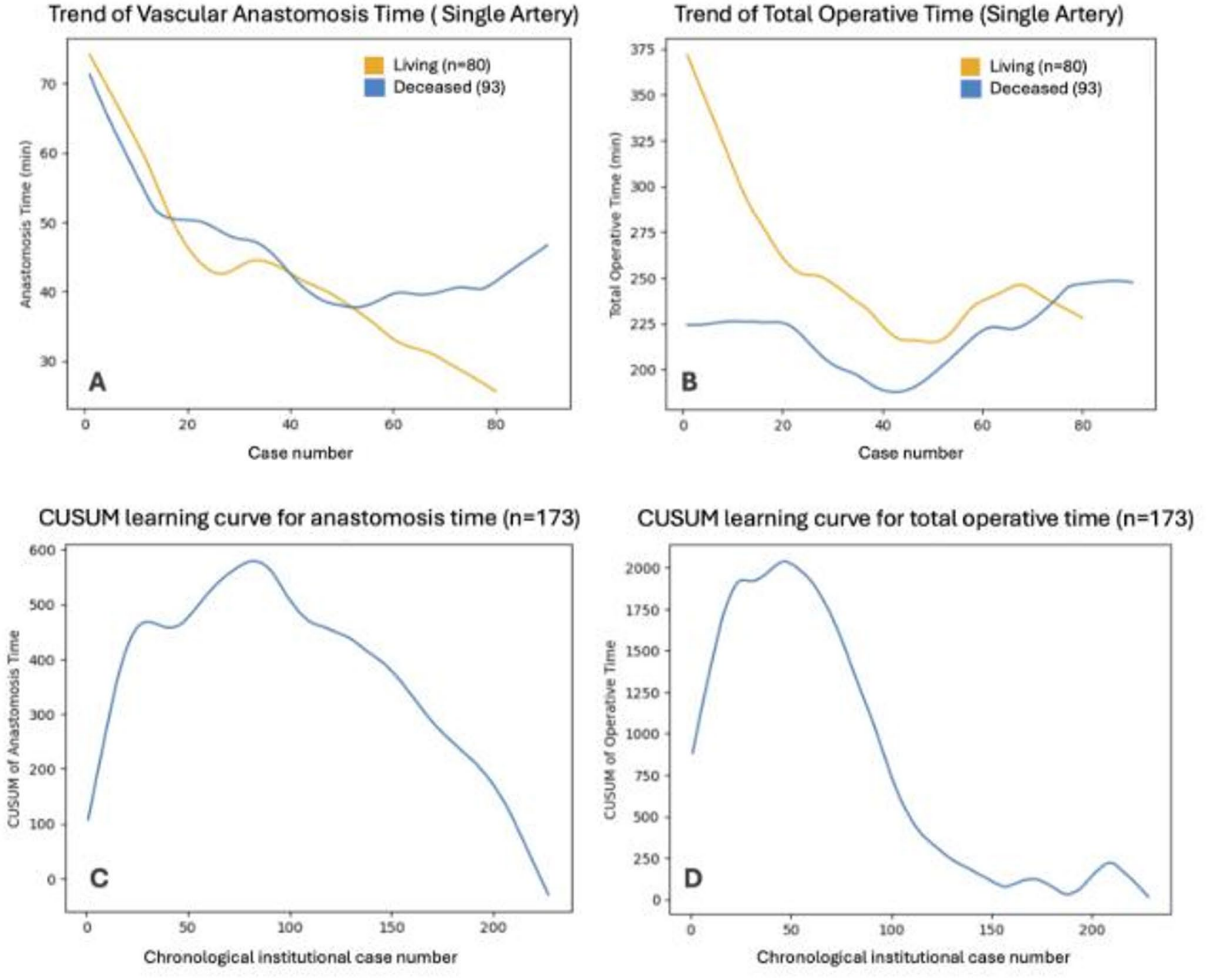
Learning curve for robot-assisted kidney transplantation in recipients with a single renal artery using locally estimated scatterplot smoothing (LOESS) and cumulative sum (CUSUM) analysis. (**A**) Trend of vascular anastomosis time in living-donor (n = 80) and deceased-donor (n = 93) recipients. (**B**) Trend of total operative time in living-donor (n = 80) and deceased-donor (n = 93) recipients. (**C**) CUSUM learning curve for vascular anastomosis time (n = 173). (**D**) CUSUM learning curve for total operative time (n = 173). Panels A and B are stratified by donor type. Panels C and D include only single-artery recipients with complete operative and vascular anastomosis time data. The x-axis represents the chronological institutional case number. Dual kidney transplantation (n = 6), simultaneous kidney-pancreas transplantation (n = 4), and cases with missing operative or vascular anastomosis time data were excluded

Learning curve analysis is summarized in Fig. 3.LOESS trend curves demonstrate a progressive improvement in operative and vascular anastomosis times, with a rapid early improvement phase within the first 35–40 cases followed by more gradual improvements. CUSUM analysis demonstrated a learning curve effect for both outcomes, with inflection points occurring at ~ 50 cases for total operative time and ~ 80 cases for anastomosis time.

## Discussion

In this study, we present one of the largest single-center experiences of RAKT, encompassing living and deceased donor transplants with single-port and multiport robotic approaches. Prior reports of RAKT have primarily focused on living donor cohorts, with a smaller proportion of DDKT [9, 10, 12–14, 17–19]. Our series describes the development of a diverse RAKT program and extends the existing literature by presenting outcomes in a cohort that better reflects the broader kidney transplant population [25, 26].

More than half of our transplanted kidneys (54.8%) were from deceased donors. Our ability to offer deceased donor RAKT hinges on several factors—a dedicated transplant operating room that was adapted to accommodate surgical robots, kidney transplant-specific surgical staff experienced in use of the robot present even on the weekends, and institutional access to multiple robots which are able to be moved from room to room. These factors underlie the importance of institutional buy-in and logistical support in expanding RAKT.

Our series is also notable for its progressive case complexity. Our initial transplants were largely limited to grafts with one artery and one vein and recipients without arterial calcifications. By 2025, approximately 23% of recipients overall had some degree of calcifications on preoperative CT. Arterial calcifications increase the risk of suboptimal clamp occlusion, dissection during arteriotomy, and increase the technical difficulty of sewing the anastomosis. To overcome this, we developed modifications to control calcified arteries, including placing additional bulldog clamps, an intracorporeal Rummel tourniquet, or placement of a laparoscopic Satinsky clamp through an additional 12-mm assistant port. In our cohort, 16.2% of grafts had multiple renal arteries, which were managed with separate anastomoses, use of a common aortic patch in.

appropriate deceased donor kidneys, or bench reconstruction with or without third-party vascular grafts. In the living-donor cohort, one recipient required a cadaveric iliac vein conduit to extend a short right renal vein, while another underwent reconstruction with a third-party external iliac artery graft to join two small donor arteries to a common orifice. In deceased-donor transplantation, vascular grafts were primarily used for arterial reconstruction in cases with short donor vessels following procurement injury or to join multiple renal arteries together to perform a single arterial anastomosis. Finally, the cohort included six dual kidney and four simultaneous, kidney/pancreas transplants demonstrating that RAKT can be applied to higher-complexity solid organ transplants [11, 15, 27].

A further distinguishing feature is our use of both single-port and multiport robotic platforms. With 70 cases, we believe this to be the largest institutional experience with single-port RAKT [20, 21, 28, 29]. This allows robotic platform selection based on donor type, recipient comorbidities, prior abdominal surgeries, and other anatomic considerations.

The median BMIs in our cohort were 33.4 kg/m² in DDKT and 30.3 kg/m² in LDKT, with over 30% of overall recipients transplanted with a BMI greater than 35 kg/m². Obesity is a well-recognized risk factor for wound complications, slower postoperative recovery, and inferior perioperative outcomes [3–5]. RAKT may expand access to kidney transplantation for obese recipients at experienced centers; however, its impact on transplant access requires further investigation [3, 5, 18].

For the overall cohort, the median operative time was 3 h 52 min, and the median vascular anastomotic time was 42 min. These operative and vascular anastomotic times are within the ranges reported for robotic and open kidney transplantation in the literature [9, 10, 15, 27]. Our times improved progressively with experience (Fig. 3). LOESS trend curves demonstrated rapid early improvement in operative efficiency within the first 35–40 cases, however, procedural proficiency as demonstrated by CUSUM analysis was achieved later, particularly for vascular anastomosis, at approximately 80 cases. In our most recent cases, DDKT anastomosis time paradoxically increased, which may be due to increasing case complexity, greater trainee involvement and the introduction of a second primary surgeon. Other centers may encounter similar factors as they introduce and expand their own RAKT programs.

Postoperatively, our median lengths of hospital stay were relatively short—two days for LDKT recipients and four days for DDKT recipients. This could reflect the benefits of minimally invasive surgery, including reduced postoperative pain and faster recovery [7, 24, 25], , as well as infrastructure that allows for close outpatient monitoring. Three-month and one-year graft function in our cohort are comparable to national averages [30]. Similarly, death-censored graft loss and 1-year mortality rates are comparable to those reported in contemporary RAKT series [9, 11, 13, 17].

One notable finding was the 24.8% ICU admission rate among our DDKT recipients. These admissions were predominantly for hemodynamic instability and respiratory distress. While the volume status of dialysis-dependent patients can affect these factors, there is plausibly a physiologic influence of the pneumoperitoneum and Trendelenburg positioning used in transperitoneal RAKT. ICU admission could also reflect increased caution exercised by surgery and anesthesia providers adapting to a newer surgical approach. ICU stays were generally short (median one day). However, this trend warrants close evaluation in future studies.

This study has several limitations, including its retrospective, single-center design, potential selection bias, and the absence of an open kidney transplantation comparator. The cohort was heterogeneous, including both living- and deceased-donor transplantation, use of single-port and multi-port robotic platforms, and increasing procedural complexity over time. As the robotic transplant program evolved, changes in institutional experience, surgical team composition, and patient selection may have introduced era effects influencing perioperative outcomes. In addition, more than half of the cohort underwent transplantation during the final year of the study, resulting in relatively short follow-up and limiting assessment of long-term graft outcomes. Finally, these findings reflect the experience of a high-volume robotic transplant center and may not be generalizable to institutions without similar robotic expertise, transplant infrastructure, and multidisciplinary support. Future studies should include comparative analyses with open kidney transplantation, as well as multivariable analyses to identify independent predictors of perioperative and graft outcomes.

## Conclusion

This experience demonstrates that RAKT can produce good allograft outcomes while expanding robotic platforms and progressively increasing donor and recipient complexity. Careful patient selection, structured development, and institutional support remain essential for success.

## Data Availability

The data that support the findings of this study are not publicly available because they contain information that could compromise the privacy of research participants. De-identified data may be made available from the corresponding author upon reasonable request and with appropriate institutional approvals.

## Abbreviations

BMI: Body mass index
CIT: Cold Ischemia time
DDKT: Deceased donor kidney transplant
DGF: Delayed graft function
DBD: Donation after brain death
DCD: Donation after circulatory death
ESRD: End-stage renal disease
IQR: Interquartile range
MP: Multi-port
POD: Post-operative day
RAKT: Robot-assisted kidney transplantation
SP: Single-port
VAS: Visual analogue scale
